# A digital twin approach for experimental acoustic hologram optimization

**DOI:** 10.1038/s44172-024-00160-0

**Published:** 2024-01-11

**Authors:** Tatsuki Fushimi, Daichi Tagami, Kenta Yamamoto, Yoichi Ochiai

**Affiliations:** 1 Institute of Library, Information and Media Science, University of Tsukuba, Kasuga Campus Kasuga 1-2, Tsukuba, 305-8550 Ibaraki Japan; 2 R&D Center for Digital Nature, University of Tsukuba, Kasuga Campus Kasuga 1-2, Tsukuba, 305-8550 Ibaraki Japan; 3 Graduate School of Comprehensive Human Sciences, University of Tsukuba, Kasuga Campus Kasuga 1-2, Tsukuba, 305-8550 Ibaraki Japan; 4 Pixie Dust Technologies, Inc., Misakicho 2-20-5, Chiyoda, 101-0061 Tokyo Japan

**Keywords:** Engineering, Acoustics

## Abstract

The need for the accurate generation of acoustic holograms has increased with the prevalence of the use of acoustophoresis methods such as ultrasonic haptic sensation, acoustic levitation, and displays. However, experimental results have shown that the actual acoustic field may differ from the simulated field owing to uncertainties in the transducer position, power and phase, or from nonlinearity and inhomogeneity in the field. Traditional methods for experimentally optimizing acoustic holograms require prior calibration and do not scale with the number of variables. Here, we propose a digital twin approach that combines feedback from experimental measurements (such as a microphone and an optical camera) in the physical setup with numerically obtained derivatives of the loss function, using automatic differentiation, to optimize the loss function. This approach is number of transducers times faster and more efficient than the classical finite difference approach, making it beneficial for various applications such as acoustophoretic volumetric displays, ultrasonic haptic sensations, and focused ultrasound therapy.

## Abstract (web summary)

Fushimi and colleagues report a digital twin based method to correct the discrepancies between simulation and experimental states for acoustic hologram optimization. This method reduces the number of experimental measurements needed, thus more efficient as compared to classical finite difference based approaches.

## Introduction

The acoustic hologram is a two-dimensional encoding of a three-dimensional acoustic field and encodes the complex wavefront via amplitude and phase specification at each point in the field. Recent advances in mid-air ultrasonics, such as ultrasonic haptic sensation^1,2^, acoustic levitation^3–5^, and acoustic streamings^6,7^, along with display technologies^8–11^, have heightened the demand for precise acoustic holograms capable of generating multiple foci from a single device. A number of acoustic hologram optimization techniques have been proposed, including Gerchberg-Saxton^12–14^, Eigensolver and Tikhonov-regularization^2^, machine learning methods^15,16^, direct solvers^17^, and greedy-type solvers^18^. In 2021, we demonstrated an automatic differentiation approach to acoustic hologram optimization^19,20^ and exhibited good accuracy with the application of automatic differentiation and the Adam optimizer in simulation.

While these numerical approaches use simulated values to optimize acoustic fields, a number of experimental results suggest that the acoustic field in reality is offset from the numerically simulated field^3,8,21–23^. These offsets could emerge from simple uncertainties in the transducer position, power, and phase, or could emerge from non-linearity, inhomogeneity, or the existence of other scatterers in the field. Recent advances in computational modeling have begun to enable the inclusion of complex nonlinear fields produced by acoustic holograms^24^, or complex fields with scatterers in the field^9,25^. However, it is still computationally expensive and cumbersome to include nonlinearity, and experimental deviations are susceptible to minor changes in the environment. This renders the attainment of experimentally accurate gradients; a challenging endeavor.

Some attempts have been made to experimentally optimize acoustic holograms^8,23,26^. For example, the offset of equilibrium points could be addressed by calibrating the focal points with the equilibrium position of the levitated particles^26^, or by the combination of a gradient descent algorithm and experimentally obtained finite differences^8^. While these optimizers are effective in achieving their targets, they require prior calibration, or experimental finite differences that do not scale well with the number of variables. These experimental deviations are known to cause performance degradation in the practical applications of acoustic holograms^8,22,24^, and there is an increasing need for better and more efficient approaches to optimize acoustic holograms in experiments. This will ultimately help to improve the haptic quality in ultrasonic tactile displays, improve the graphic generation capabilities of acoustophoretic volumetric displays, and improve the positioning accuracy in the potential application of acoustic levitation. Where the current system needs to accept the experimental deviation or collect a significant amount of data to collect the deviation; this method has the potential for application in practice.

**Fig. 1:**
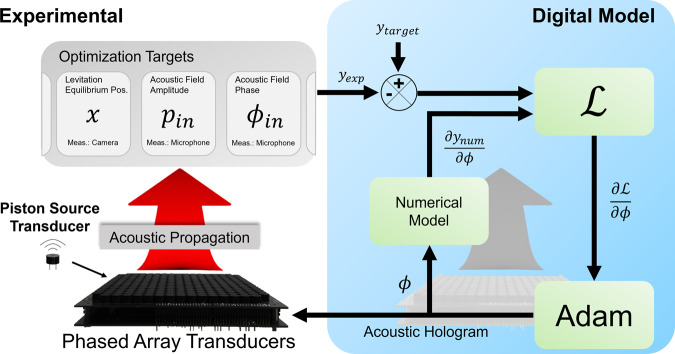
In-situ optimization with digital twin. Acoustic hologram, i.e. the phase delay specification for each transducer is passed on to both the experimental setup and numerical model. Both propagate the hologram in the experiment and numerical simulation. The optimization targets can be anything that can be physically measured and modeled. The experimental measurements can be made via various instruments such as cameras, microphones, or laser Doppler vibrometers. After obtaining the experimental measurements, the difference between the experimental measurements (*y*_exp_) and target (*y*_target_) is fed into the loss function along with the derivative of the numerical model ($\frac{\delta {y}_{{\rm {num}}}}{\delta \phi }$) determined via automatic differentiation. The solutions are then updated accordingly using stochastic gradient descent algorithms.

Herein, we propose a digital twin approach for optimizing the acoustic holograms, as shown in Fig. 1, with the aim of minimizing the difference between the target and experimental states with a minimal number of measurements per step. A digital twin serves as a comprehensive virtual model designed to accurately emulate a corresponding physical entity. Recently, this concept has been extended to the field of acoustics as a tool to enhance structural health monitoring in engineering systems^27–29^. Experimental measurements in situ (physical setup) can be fed back into the loss function of the optimizer, and the digital model of the experimental setup is then used to obtain the gradient of the loss function with respect to each variable using automatic differentiation. Because the gradient of the loss function is approximated numerically from the digital model, there is no need for the experimental finite difference algorithm. Thus, the optimizer will complete its optimization at least the “number of variables” times faster than the classical finite difference approach. Digital twin optimization is highly beneficial in phased array transducers (PAT) applications because the number of transducers is in the magnitude of 10^2^–10^3^ (i.e. up to 10^3^ times faster). Considering the fact that the optimization is performed iteratively, this causes a significant enhancement of the performance and efficiency. Such methods that are used to connect experimental to digital models have been proven to be effective in machine learning^30^ and optics^31^; the application of this method in the acoustic hologram could be beneficial in the practical application of acoustics. In addition, we present insights into digital twin optimization such as “experimental optimization of iteratively calculated variables” and “design principles of loss functions in the experimental optimization with digital twin”, which are highly relevant in machine learning and the optics field as well.

The core of the optimization algorithm is the Diff-PAT; an acoustic hologram optimization method based on automatic differentiation demonstrated by Fushimi et al.^19^. The initial guess of the acoustic hologram is updated iteratively using the Adam optimizer based on the differentiated loss value with respect to each phase of the transducers. Given the loss function, *L*_*t*_(*θ*_*t*_), the Adam optimizer iteratively updates the initial guess of the variables (*θ*_*t*_) by

1 $${\theta }_{t}={\theta }_{t-1}-\alpha \cdot \frac{{\hat{m}}_{t}}{\sqrt{{\hat{v}}_{t}}+\epsilon }$$

where *θ* is the optimization variable, and subscript *t* is the step number. ${\hat{v}}_{t}=\frac{{v}_{t}}{1-{\beta }_{2}^{t}}$, ${\hat{m}}_{t}=\frac{{m}_{t}}{1-{\beta }_{1}^{t}}$, ${v}_{t}={\beta }_{2}\cdot {v}_{t-1}+(1-{\beta }_{2})\cdot {g}_{t}^{2}$, *m*_*t*_ = *β*_1_ ⋅ *m*_*t*−1_+(1−*β*_1_) ⋅ *g*_*t*_, and ${g}_{t}=\frac{\delta {L}_{t}({\theta }_{t-1})}{\delta \theta }$. Here, *α* is the step size/learning rate, *β*_1_ = 0.9, *β*_2_ = 0.999, and *ϵ* = 1 × 10^−7^ are exponential decay rates for the moment estimates. We adjusted the learning rates depending on each application, and the used value was specified within each case.

As shown above, the Adam optimizer only uses the derivative of the loss function (*L*_*t*_(*θ*_*t*−1_)) to update the parameters. Naturally, the question arises on “how can it know which way to descend to in the experiment when it only uses a gradient that is numerically obtained?” In a nutshell, we design the loss function such that “the experimentally obtained states carry over to the derivative of the loss function”, and when it does, “the gradient near the target state is steep”. Thus, when the loss function is properly designed; the Adam optimizer determines the minima at the target state in the experiments.

For example, a loss function could be specified as ${L}_{t}({\theta }_{t-1})={(T-{f_{{\rm {exp}}}}({\theta }_{t-1}))}^{2}$, where *T* is the target value (i.e. target acoustic pressure, phase, or equilibrium position), and subscript “exp” means experimentally obtained. We define the gradient to be given by $\frac{\delta {L}_{t}}{\delta {\theta }_{t}}=-2(T-{f_{{\rm {exp}}}}({\theta }_{t-1}))\frac{\delta {f}_{{\rm {num}}}({\theta }_{t-1})}{\delta \theta }$ with subscript “num” denoting a numerically obtained value. Thus, the experimental optimum point naturally becomes the destination of the Adam optimizer. The optimization target can be anything that can be physically measured and predicted in in-situ optimization. In this study, we demonstrate the optimization of the (i) acoustic pressure and (ii) equilibrium position to demonstrate the relevance of digital twin optimization in PAT and acoustic holograms in general. Finally, we discuss the design of the loss function in the discussion section.

First, we describe digital twin optimization for the acoustic pressure field. We use PAT as described in the “Methods” section, and it takes phase-only acoustic holograms (operates in phase-only A mode hologram). As in Fushimi et al.^20^, we begin by defining a suitable loss function for the phase-only (A:i), amplitude-only (A:ii), and phase and amplitude (A:iii) optimization:

2 $$L({\phi }_{t})=\left[{({A}_{{\rm {c}}}\cos {\phi }_{{\rm {c}}}-{A}_{{\rm {p}}}^{{\rm {exp}}}\cos ({\phi }_{{\rm {p}}}^{{\rm {exp}}}))}^{2}+{({A}_{{\rm {c}}}\sin {\phi }_{{\rm {c}}}-{A}_{{\rm {p}}}^{{\rm {exp}}}\sin ({\phi }_{{\rm {p}}}^{{\rm {exp}}}))}^{2}\right],$$

where *A*_c_ and *ϕ*_c_ are the target pressure amplitude and phase, and ${A}_{{\rm {p}}}^{{\rm {exp}}}(x,{x}_{t},{\phi }_{t})=| {p}_{{\rm {in}}}(x,{x}_{t},{\phi }_{t})| +G({p}_{{\rm {exp}}}-| {p}_{{\rm {in}}}(x,{x}_{t},{\phi }_{t})| )$ and ${\phi }_{{\rm {p}}}^{{\rm {exp}}}=\arg ({p}_{{\rm {in}}}(x,{x}_{t},{\phi }_{t}))+G({\phi }_{{\rm {exp}}}-\arg ({p}_{{\rm {in}}}(x,{x}_{t},{\phi }_{t})))$ are the substituted pressure amplitude and phase, respectively. The substituted pressure amplitude allows the automatic differentiation package (in this case TensorFlow) to track the gradient of the function, whereas the inside of function *G*() is untracked by the package (achieved by e.g. *tf.stop_gradient()* in TensorFlow). We note that this process does not act as mathematical operators, and only serves a functional purpose to introduce foreign variables to the computational graph of TensorFlow. As in Fushimi et al.^20^, *A*_c_ = 1 was set for A:i and *ϕ*_c_ = 0 was set for A:ii.

The experimentally obtained values (such as *p*_exp_ and *ϕ*_exp_) are obtained using a calibrated pressure microphone (B&K Type 4138-A-015, pressure sensitivity ${p}_{{\rm {sens}}}^{{\rm {mic}}}=1.0\,{{{{{{{\rm{mV}}}}}}}}\,{{{{{{{{\rm{Pa}}}}}}}}}^{-1}$), as detailed in the “Methods” section. Twenty target phases and amplitudes were set with a constant focal point, *x* = (0, 0, 0.04) m. The phase linearly increased from 0 to 2*π*, and the amplitude increased linearly from 10% to 90% of *p*_max_. *p*_max_ was set as the pressure amplitude with a single focus point in the numerical simulation. The maximum iteration number was set to 100. The experimental measurements and optimizations were repeated three times (*S* = 3) to obtain the mean and standard deviation of the pressure and amplitude. The statistical analysis was performed on Matlab R2022a. The mean phase was obtained by ${\phi }_{{\rm {mean}}}=\arctan \left(\frac{\mathop{\sum }\nolimits_{s}^{S}\sin ({\phi }_{{\rm {meas}}}^{s})}{\mathop{\sum }\nolimits_{s}^{S}\cos ({\phi }_{{\rm {meas}}}^{s})}\right)$ where *ϕ*_meas_ is the measured phase.

We can also apply the in-situ digital optimization for the equilibrium position of the levitated particle. While the acoustic pressure field can be calculated easily using Huygens’ approach, the determination of the equilibrium position requires the balancing of the acoustic radiation force and gravity. This calculation requires a root-finding algorithm that iteratively updates its guess. Here, we use the single-axis acoustic levitator (2 SonicSurface arrays separated by a distance of 0.215 m). The target equilibrium shape was set as a circle with radius *r* = 3 mm, where *n*th focal point is calculated by ${x}_{f}^{t,n}=(0,r\sin (\pi +\frac{2\pi n}{29}),0.0119+r+r\cos (\pi +\frac{2\pi n}{29}))$, with *n* ∈ {0, 1, …, 28}. While it is possible to optimize the equilibrium position from the acoustic hologram as in pressure field optimization, the levitation conditions are not necessarily guaranteed for all possible phase combinations. Thus, the optimization variable was constrained to known stable solutions using a single focal point and twin trap^4^:

3 $${\phi }_{t}={\phi }_{{\rm {focal}}}+{\phi }_{{\rm {twin}}},$$

where ${\phi }_{{\rm {focal}}}=-\frac{2\pi {f}_{0}}{{c}_{0}}[{\rm {d}}({{{{{{{{\boldsymbol{x}}}}}}}}}_{{{{{{{{\boldsymbol{f}}}}}}}}}^{{{{{{{{\boldsymbol{t}}}}}}}},{{{{{{{\boldsymbol{n}}}}}}}}},{{{{{{{{\boldsymbol{x}}}}}}}}}_{{{{{{{{\boldsymbol{t}}}}}}}}})-{\rm {d}}({x}_{o},{{{{{{{{\boldsymbol{x}}}}}}}}}_{{{{{{{{\boldsymbol{f}}}}}}}}}^{{{{{{{{\boldsymbol{t}}}}}}}},{{{{{{{\boldsymbol{n}}}}}}}}})]$, and *ϕ*_twin_ are 0 and *π* for the bottom and upper arrays, respectively. *x*_0_ is the origin (0,0,0) of the array which is the centre of the 16 by 16 PAT, and on the surface level of the transducers. The single focus is calculated such that the acoustic signal from each transducer meets at the focal point simultaneously. As in a previous study^8^, the target shape was specified using the focal points, and the equilibrium positions were recorded (waited 2.5 s after sending the commands to PAT for the steady state) using optical methods as described in the “Methods” section.

For a spherical particle in the Rayleigh regime (*r* << *λ*), the acoustic radiation force was calculated using Gor’kov^32,33^:

4 $$\left[\begin{array}{c}{F}_{x}\\ {F}_{y}\\ {F}_{z}\end{array}\right]=-\frac{4\pi }{3}{a}^{3}\bigtriangledown \left[\frac{1}{2}{{{{{{{\rm{Re}}}}}}}}[\,{f}_{1}]{\kappa }_{0}\langle {p}_{{\rm {in}}}{(x,{\phi }_{t})}^{2}\rangle -\frac{3}{4}{{{{{{{\rm{Re}}}}}}}}[\,{f}_{2}]{\rho }_{0}\langle {v}_{{\rm {in}}}{(x,{\phi }_{t})}^{2}\rangle \right]$$

where ${f}_{1}=1-\tilde{\kappa }$ and ${f}_{2}=\frac{2(\widetilde{\rho }-1)}{2\widetilde{\rho }}$. $\tilde{\kappa }=\frac{{\kappa }_{p}}{{\kappa }_{0}}$ and $\widetilde{\rho }=\frac{{\rho }_{{\rm {p}}}}{{\rho }_{0}}$. Subscript 0 and p represent the surrounding media and particle property, and $\kappa =\frac{1}{\rho {c}^{2}}$ where *ρ* and *a* are the density and radius of the sphere, respectively. ${v}_{{\rm {in}}}(x,{\phi }_{t})=\Big| \bigtriangledown \left(\frac{{p}_{{\rm {in}}}}{{\rho }_{0}\omega i}\right)\Big|$ is the acoustic velocity field. Moreover, the particle properties were arbitrarily set as *a* = 0.7 mm, *ρ*_*p*_ = 40 kg m^−3^, and *c*_p_ = 900 m s^−1^.

To determine the numerical equilibrium position, a root-finding algorithm (Newton gradient descent) was used^26^:

5 $${{{{{{{{{\bf{x}}}}}}}}}_{{{{{{{{\bf{e}}}}}}}}}}^{n+1}={{{{{{{{{\bf{x}}}}}}}}}_{{{{{{{{\bf{e}}}}}}}}}}^{n}-{J}^{-1}\left[{F}_{x},{F}_{y},{F}_{z}^{g}\right]$$

where $$J=\left[\begin{array}{c}\frac{\delta {F}_{x}}{\delta x}\frac{\delta {F}_{x}}{\delta y}\frac{\delta {F}_{x}}{\delta z}\\ \frac{\delta {F}_{y}}{\delta x}\frac{\delta {F}_{y}}{\delta y}\frac{\delta {F}_{y}}{\delta z}\\ \frac{\delta {F}_{z}^{{\rm {g}}}}{\delta x}\frac{\delta {F}_{z}^{{\rm {g}}}}{\delta y}\frac{\delta {F}_{z}^{{\rm {g}}}}{\delta z}\end{array}\right]$$, ${F}_{z}^{{\rm {g}}}={F}_{z}-mg$, *x*_e_ is the Jacobian matrix, total *z* force, and equilibrium position, respectively. The root-finding algorithm was executed until the delta between the current and the previous step was below 0.1 mm.

At this point, the numerical model can be integrated into the digital twin; however, this model is computationally very expensive. Inspecting the calculated equilibrium position reveals that the mapping from the focal to equilibrium point is simple^26^, and as simple as two sets of polynomial functions. To fit the polynomial functions to the equilibrium position, the equilibrium positions in the region of interest (ROI); −*λ* ≤ *y* ≤ *λ* and −*λ* + *r*_c_ ≤ *z* ≤ *λ* + *r*_c_ were calculated with a step size of $\frac{\lambda }{5}$. *r*_c_ = 0.119 is the vertical offset of the numerical simulation to set the ROI. Then, the polynomial function was fitted to the data set using the Matlab curve fitting toolbox (version 3.7); ${x}_{e}^{y}={a}_{0}+{a}_{1}{x}_{f}^{y}+{a}_{2}{x}_{f}^{z}$ where *a*_0_,*a*_1_ and *a*_2_ are 7.367 × 10^−12^, 0.9981, and −6.962 × 10^−10^, respectively. ${x}_{e}^{z}={b}_{0}+{b}_{1}{x}_{f}^{y}+{b}_{2}{x}_{f}^{z}+{b}_{3}{({x}_{e}^{y})}^{2}+{b}_{4}{x}_{e}^{y}{x}_{e}^{z}$ where *b*_0_, *b*_1_, *b*_2_, *b*_3_, *b*_4_ are −1.524 × 10^−4^, −2.934 × 10^−8^, −1.000, −1.143 × 10^−2^, and −2.343 × 10^−7^. The *r*-squared goodness of fit was 0.999 for both cases.

This significantly simplifies the numerical model, guarantees stability within the ROI, and reduces the number of optimization variables. Similarly to the pressure field optimization, the loss function was set as $L=\sqrt{{({y}_{{\rm {c}}}-{y}_{{\rm {p}}}^{{\rm {exp}}})}^{2}+{({z}_{{\rm {c}}}-{z}_{{\rm {p}}}^{{\rm {exp}}})}^{2}}$ where *y*_c_ and *z*_c_ are the target positions in the *y* and *z* axes. ${y}_{{\rm {p}}}^{{\rm {exp}}}={x}_{e}^{y}({x}_{f}^{y},{x}_{f}^{z})+G({y}^{{\rm {exp}}}-{x}_{e}^{y}({x}_{f}^{y},{x}_{f}^{z}))$ and ${z}_{p}^{{\rm {exp}}}={x}_{e}^{z}({x}_{f}^{y},{x}_{f}^{z})+G({z}^{{\rm {exp}}}-{x}_{e}^{z}({x}_{f}^{y},{x}_{f}^{z}))$ are the substituted experimental equilibrium points. The maximum iteration number was set to 25.

## Results and discussion

### Pressure field optimization

**Fig. 2:**
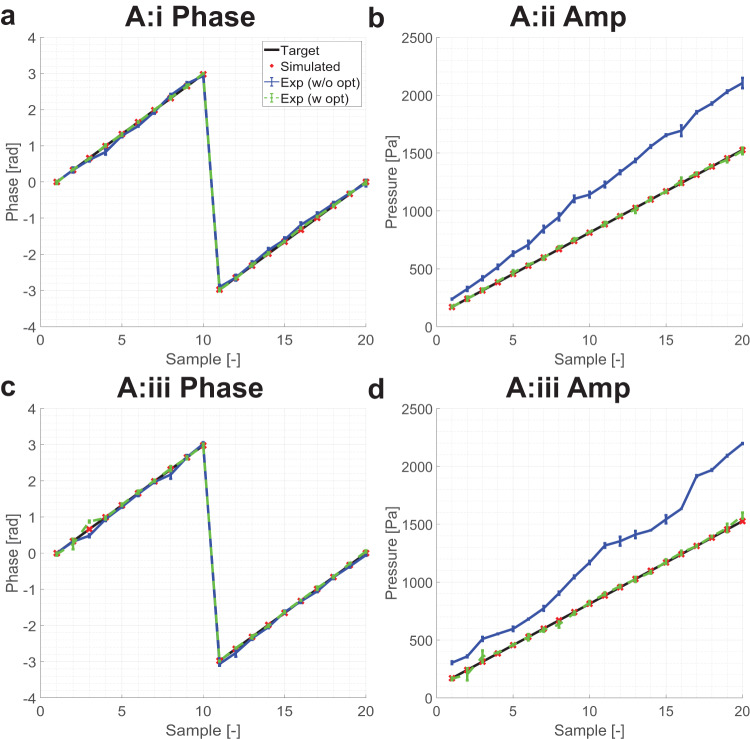
Comparison of pressure field with various test conditions. Numerical optimization only, experimental measurements of the numerically optimized solution, and experimentally optimized value are shown by a red `x', a blue line with standard deviation, and a green dotted line with standard deviation (sample size of 3), respectively. **a** A:i (phase only hologram with target phase optimization) configuration with its phase performance, **b** A:ii (phase only hologram with target amplitude optimization) configuration with its amplitude performance, **c** A:iii (phase only hologram with target amplitude and phase optimization) configuration with its phase performance and **d** amplitude performance in A:iii configuration. The black line indicates the target for each instance.

The results for the A:i, A:ii, and A:iii optimizations are as shown in Fig. 2a, b, and c, d, respectively. As shown in Fig. 2a–d, the phased array perfectly achieves target-optimized states in optimal conditions, i.e. numerical simulation (red crosses). The performance of the optimizer in the numerical simulation is evaluated by the mean square of error (MSE) for both phases and amplitude. The MSE phases are 3.16 × 10^−13^ and 3.17 × 10^−13^ for A:i and A:iii, respectively. The amplitude error is also low with 1.79 × 10^−6^ and 2.50 × 10^−6^ for A:ii and A:iii, respectively.

The numerically optimum solution works well for target phase optimization in experiments, and the experimental value closely achieves the target as shown in Fig. 2a and c. The experimental phase error, measured in MSE are 5.43 × 10^−3^, and 4.81 × 10^−3^ for A:i and A:iii, respectively. The employment of the experimental optimization reduces the error to 3.23 × 10^−4^ and 3.08 × 10^−3^, respectively, for A:i and A:iii. However, because the numerical optima performs well in the first place, the improvement is minor and the error reduction rate (measured by the ratio between experimental and optimized MSE) is only approximately 16.8 and 1.56 for A:i and A:ii, respectively.

However, the numerically obtained optimum solution does not apply well for amplitudes in the experimental condition as shown in Fig. 2b and d. The pressure amplitude error measured in MSE is 1.41 × 10^5^ and 1.46 × 10^5^ for A:ii and A:iii, respectively, in the experiment. By the employment of experimental optimization, the pressure error reduces to 89.2 and 423 for A:ii and A:iii, respectively. The A:iii optimizer consistently performs worse than the counterparts such as A:i or A:ii, and this is attributed to the fact that the loss function is more complex than optimizing for either parameter. The error reduction rate is 1580 and 345 times for A:ii and A:iii, respectively. The results are summarized in Table 1.

**Table 1:** Summary of phase and amplitude performance measured in mean squared error (MSE).

Metric	Category	Numerical	Experimental	Optimized	Improvement
Phase	A:i	3.16 × 10^−13^	5.43 × 10^−3^	3.23 × 10^−4^	16.8
Phase	A:iii	3.17 × 10^−13^	4.81 × 10^−3^	3.08 × 10^−3^	1.56
Amplitude	A:ii	1.79 × 10^−6^	1.41 × 10^5^	89.2	1580
Amplitude	A:iii	2.5 × 10^−6^	1.46 × 10^5^	423	345

Improvement measured by taking the ratio between experimental and optimized MSE.

**Fig. 3:**
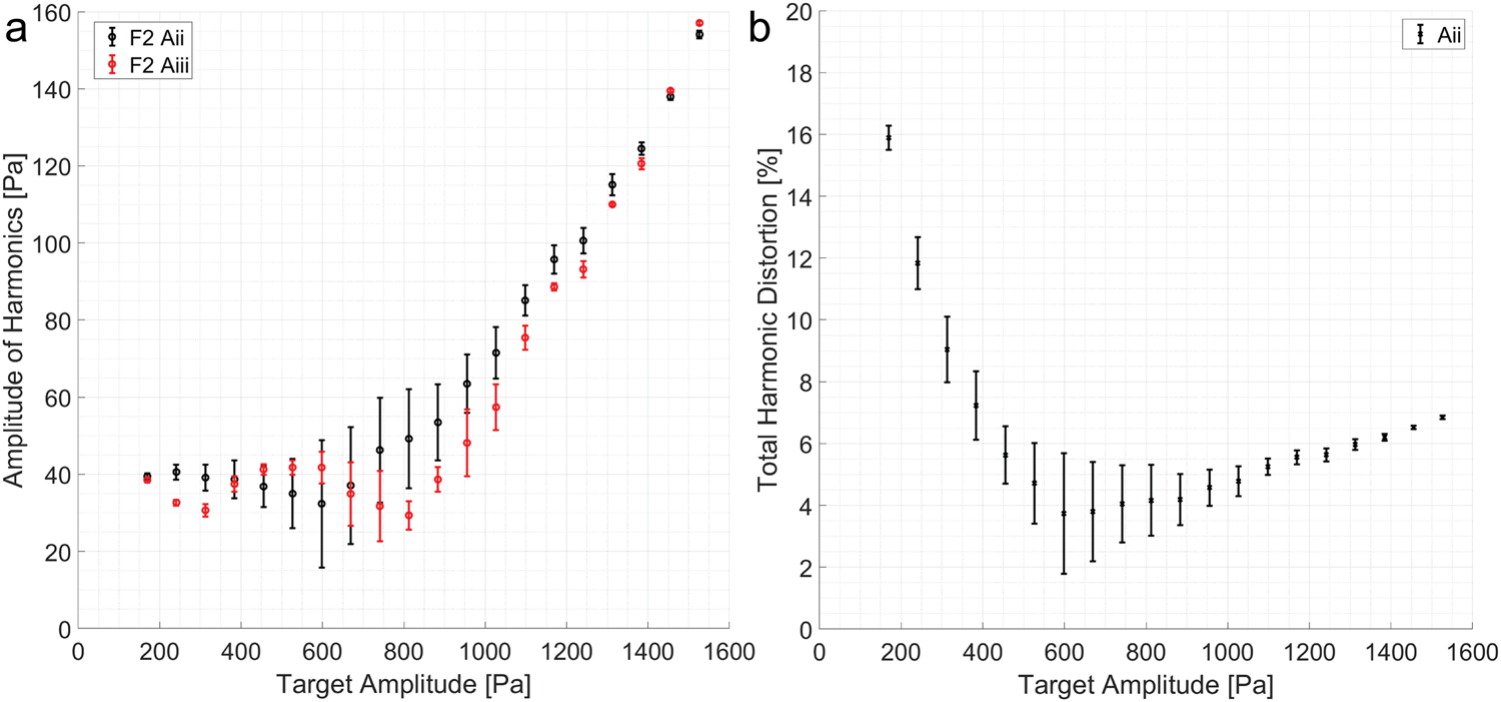
Investigation of nonlinear effects in pressure field optimization. The error bar shows the standard deviation with a sample size of 3. **a** Shows the amplitude of higher harmonics generation for each target amplitudes in Fig. 2. The black and red points indicate results from A:ii and A:iii, respectively. The circles indicate the second (80 kHz) harmonics. **b** Shows the total harmonic distortion for each target amplitude. It starts high but settles to ≈6% after the target amplitude of 1200 Pa.

One of the potential causes of the experimental deviation is the nonlinearity of the field. Nonlinearity of the field is not only an issue in mid-air ultrasound but also from measurements to the practical application of audible acoustics in mid-air^34,35^. The generation of higher harmonics has been discussed as a potential issue by Andrade et al.^21^ and it has also been reported to cause issues in underwater acoustics^24^. Figure 3 shows the measured nonlinearity from the non-optimized field, and Fig. 3a shows that the second harmonics generation (F2) grows as the target amplitude increases. However, the total harmonic distortion ($\frac{\sqrt{({F}_{2}^{2})}}{{F}_{1}}$) decreases with the increased target amplitude. Thus, while the nonlinear effects are present, it does not fully explain the experimental deviation.

Despite the nonlinearity and unknown cause of the experimental deviation, the digital twin optimizer still determines the acoustic holograms for the desired outcome. The in-situ optimization of the pressure amplitude is directly applicable in HCI applications (ultrasonic haptic sensation, displays, acoustic streaming), medical applications where the nonlinear and complex media is present in the propagation media, or additive manufacturing where scattering conditions are constantly changing. In-situ optimization can easily be scaled to implement multi-point optimization, and the benefit of in-situ optimization is enhanced with the number of optimization variables and targets. In such cases, the experimental measurements may still become the bottleneck in the optimization process, and efficient measurement methods based on optics (for e.g. schileren^36^ or the laser Doppler vibrometer^37^) may be better suited for faster optimization.

### Equilibrium point optimization

**Fig. 4:**
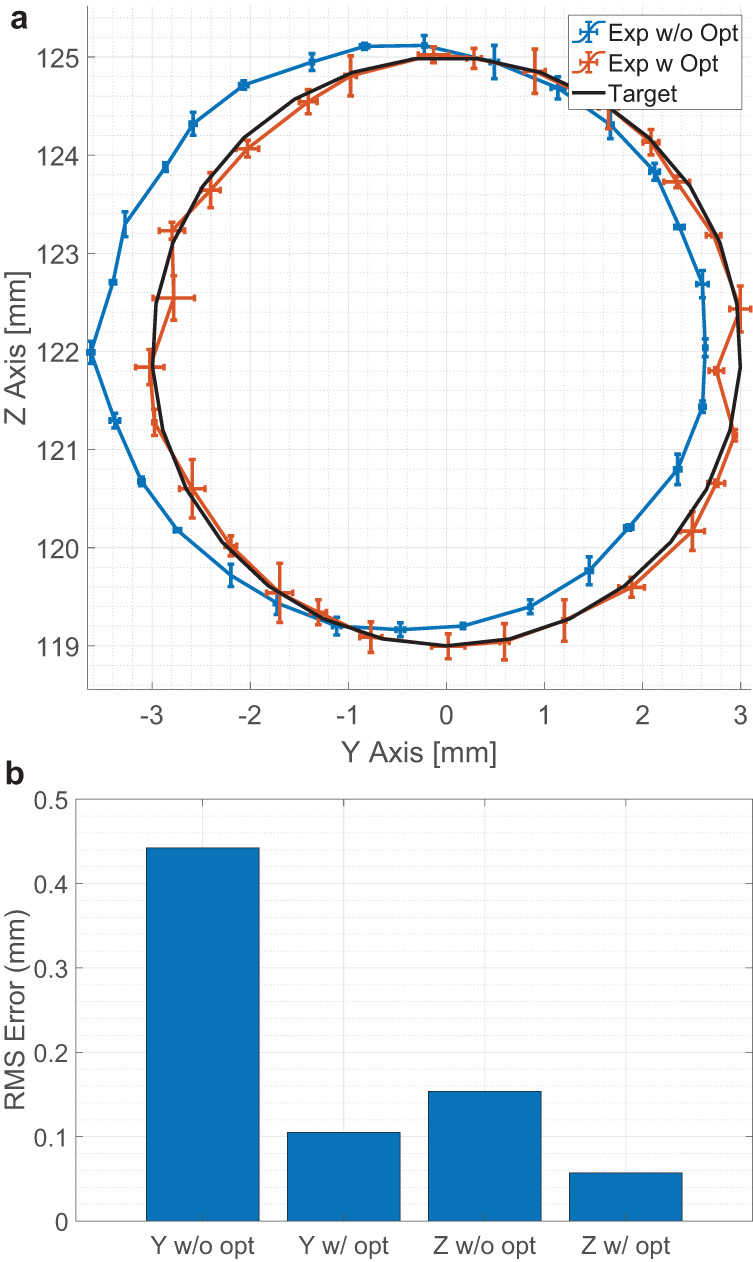
Results of equilibrium position optimization. **a** Shows the absolute position of the target (black), experimental results without optimization (blue), and with optimization (orange). The error bar indicates the standard deviation (sample size of 3). **b** Shows the root mean square (RMS) error of the trajectory for each axis with and without optimization.

The results are as shown in Fig. 4, and the unoptimized focal point does not approximate the equilibrium position (root mean square error (RMS) error of 0.442 and 0.154 mm); the optimization process is required. Digital twin optimization was performed with the Adam optimizer (learning rate = 5 × 10^−4^), with the initial solution set as the target focal point (*q* = 0). For subsequent optimization (*q* ≤ 1), the initial guess was set to the optimized focal points from the last target. The optimizer was iterated for 25 steps, and the results are as shown in Fig. 4a. After the experimental optimization, the RMS error dropped to 0.105 and 0.057 mm for the *y* and *z* axes, respectively, significantly improving the positioning accuracy of the acoustic levitator, as shown in Fig. 4b.

Where previous methods^26^ required a calibration map (which typically requires many hours to measure), this in-situ optimizer achieves equivalent performance (RMS of 0.11 and 0.030 mm for horizontal and vertical axes, respectively, for methods with prior calibration^26^) without the calibration map. Thus, this could be used to improve the image quality in acoustophoretic volumetric displays or improve specimen positioning accuracy for diagnostics/analytic purposes^38–40^.

### Designing the loss function

In-situ optimization has been demonstrated previously in machine learning and optical systems; however, the design of the loss function itself has not been discussed in depth. The design of the loss function is the most critical in achieving a successful experimental optimization with the digital twin. This is because we do not identify any derivatives via experiments. The loss function needs to be designed such that the experimental values are passed on to the optimizer; otherwise, the optimizer will only find the numerical optima. This could be a potential pitfall for the future of experimental optimization with the digital twin, and we will present an example where such a design may be critical.

We set a toy maximization problem where *f*_ideal_(*x*) = −*x*^2^, and the experimental deviated function, *f*_exp_(*x*) = −(*x*−10)^2^ (see the section “Data availability” for the codes). A simple loss function to obtain the maxima of the function is; *L*_1_(*x*) = −*f*_ideal_(*x*), *L*_2_ = −*f*_exp_(*x*). Trivially, the solution is *x* = 0, and *x* = 10 for the ideal and experimental case, respectively. We then convert the loss function to include the experimental data and preserve the automatic differentiated value; *L*_3_ = −(*f*_ideal_(*x*) + *G*(*f*_exp_(*x*) −*f*_ideal_(*x*))). We solved the *L*_3_ with the Adam optimizer (learning rate = 0.05, iteration number of 3000), and obtained the mean optimal points by repeating the optimization 100 times with random initial values between -2.5 and 22.5 (i.e. center at 10).

When such optimization is performed, the function converges only to the numerical optima (*x* = 0). This is because the differentiated loss function does not carry any information regarding the experimental states, and cannot descend to the experimental maxima. Thus, for a function to be maximized, the loss function needs to have a steep gradient near the maxima, and still have a term *f*(*x*) when differentiated.

One such function is $\frac{\delta {L}_{4}}{\delta x}=\frac{{f}^{{\prime} }(x)}{f(x)}$, or ${L}_{4}(x)=\log f(x) = \log \left(\right.{f}_{{{{{{\rm{ideal}}}}}}}(x)+G({f}_{{{{{\rm{{exp}}}}}}}(x)-{f}_{{{{{{\rm{ideal}}}}}}}(x))$. This is not a perfect maximization function, because (1) the value does not reach the optima when it starts from the left-hand side of optima, (i.e. *x* ≤ 10 the returned solution has a mean of *x* = 0.300, with a s.t.d. of 1.22, number of instances 54/100) and (2) the solution could be NaN out depending on the initial value; however, the solution improves to a mean of 8.32 (std: 0.491, number of instances 41/100), given a good initial guess (*x* ≥ 10). Further study is required to identify more suitable loss functions, but this knowledge should be helpful in the future applications of in-situ optimization.

### Measurements methods

The acoustic pressure field can be measured via a wide range of methods, from the classical use of a pressure microphone, optical methods (LDV^37^, Schlieren^36^, PIV^41^), to thermal methods^12,42^. If calibration is properly performed, experimentally determined values from these methods can be directly substituted into the digital twin workflow. Thus, non-contact and remote measuring methods could be employed in the future to minimize the effect of scattering from the microphones. However, if not calibrated, it is more challenging to implement it back into the digital twin workflow, and its functions may be limited to ‘minimize’ or ‘maximize’ the pressure field with respect to the normalized values.

## Conclusion

In conclusion, we presented an in-situ optimization method of the acoustic hologram with a digital twin. This optimizer obtained the experimental measurements and optimized the hologram using experimental measurements and numerical gradients. We demonstrated two approaches for the measurements (microphone, and camera), and two approaches for the modeling (i.e. direct numerical model, and polynomial approximation). Both methods were successful in improving the performance of the hologram, and up to 1580 times improvements were recorded in terms of pressure amplitude optimization. Furthermore, the experimental optimization was performed by measuring once per step, which significantly reduced the number of measurements needed in the experimental setup. This optimization method, along with the design philosophy for the loss function will be directly helpful in improving the performance of the practical application of PAT such as acoustophoretic volumetric displays, ultrasonic tactile displays, and mid-air acoustic levitation.

## Methods

### Pressure calculation

The complex pressure at a specific point (*x*) generated by PAT is calculated by

6 $${p}_{{\rm {in}}}(x,{x}_{t},{\phi }_{t})=\mathop{\sum }\limits_{t=1}^{T}\frac{{P}_{0}}{d({{{{{{{\boldsymbol{x}}}}}}}},{{{{{{{{\boldsymbol{x}}}}}}}}}_{{{{{{{{\boldsymbol{t}}}}}}}}})}D(\eta ){{\rm {e}}}^{j(kd({{{{{{{\boldsymbol{x}}}}}}}},{{{{{{{{\boldsymbol{x}}}}}}}}}_{{{{{{{{\boldsymbol{t}}}}}}}}})+{\phi }_{t})},$$

where *P*_0_ is the transducer power at 1 m, *d*(***x***, ***x***_***t***_) is the Euclidean distance between the transducer position (*x*_*t*_) and the specified position (*x*). $D(\eta )=\frac{2{J}_{1}(kr\sin \eta )}{kr\sin \eta }$ is the directivity function for a piston source. $k=\frac{2\pi {f}_{0}}{{c}_{0}}$ is the wavenumber, with *f* = 40 kHz and *c*_0_ = 341 m s^−1^.

We employed a phased array made of 256 transducers of a 1 cm diameter, operating at 40 kHz (Manorshi, MSO-P1040H07T, *P*_0_ = *P*_v_*V*_a_, where *P*_v_ = 0.31 Pa V^−1^ at 1 m, and *V*_a_ = 5 V is the actuated voltage) and we referred to SonicSurface for details regarding the signal generation for each transducer)^43^. The transducers are arranged in a 16 × 16 square flat grid. A field-programmable gate array (FPGA) (EP4CE6E22C8N—ALTERA IV Core Board, Waveshare) generates the control signals multiplexed into 8 channels per output pin; shift registers (74HC595, TI) demultiplex the pin signal into 8 channels, and the channels get amplified by drivers (MIC4127 from MT) up to a 20 peak-to-peak voltage. The signals to be generated are sent by a computer to the FPGA using UART at 230,400 bps, enabling the update of the emission phases at 190 times per second. The phase resolution was 32 divisions per period. The transducer power coefficient was measured by taking the average of 10 transducers, and the microphone was oriented such that the microphone pointed towards the PAT.

### Experimental pressure field measurements

The calibrated microphone was connected to the conditioning amplifier (B&K Type 2690), and the output voltage was recorded using the USB oscilloscope (TiePie Handyscope HS5). The captured data were converted from voltage to pressure amplitude based on the calibration data, and the FFT was obtained to determine the amplitude and phase at the fundamental frequency (40 kHz). The reference for the phase was set as the clock signal from the FPGA board. The microphone was attached to the XYZ stage (Controller: OptoSigma SHOT-304GS, Stages: OptoSigma OSMS20-85, OSMS26-100, OSMS26-100) to accurately control the position of the microphone, as shown in the Supplementary Fig. S1. The stage commands were sent via Serial communication (baud rate = 9600) using the pyOptoSigma package (https://github.com/ken1row/PyOptoSigma). The optimization scheme was implemented in Python (ver 3.10.7) and codes to fully recreate the setup were made available as shown in the “Data availability” section (TensorFlow version 2.10.0). The Adam optimizer was used, the learning rate was 0.05, and the optimizer was iterated 100 times. Experimental measurements and optimizations were repeated three times to obtain the average performance, its standard deviation and mean squared error.

### Experimental measurement of equilibrium position

The experiment was conducted on top of an optical table (Thorlabs B90120A, SDP90120), and the equilibrium position was captured by a USB-C high-speed camera (Photron INFINICAM UC-1) with a Nikon F-to-C Mount Adaptor (Kenko Tokina) and a single-focus lens (Tamron SP AF180mm F/3.5Di), as shown in Supplementary Fig. S2. We used a parallel light source (LED Tempo, IPS-FPP150150-IF15) for background illumination, which provides uniform light intensity across its entire area. The camera then captured the particle’s silhouette to accurately determine its position. A CMM-stylus (RENISHAW A-5000-7557) was attached to the aforementioned XYZ stages to obtain both the pixel-to-mm conversion rate (1.408 × 10^−5^ mm pix^−1^) and datum point. The camera was operated through Python SDK (pypuclib (https://github.com/infinicam/pypuclib)), and the equilibrium position and camera calibration was identified using the hough circle transform on OpenCV (version 4.6.0).

### Utilization of generative AIs in manuscript

The authors employed OpenAI’s ChatGPT (GPT-4) for the generation of certain sections of the manuscript and abstracts. Subsequently, we meticulously reviewed and verified the output to ensure its accuracy and relevance to the subject matter.

### Supplementary information

Supplementary Material

## Data Availability

The data that support the findings of this study (experimental data) are openly available in Zenodo (10.5281/zenodo.10065462) and in supplementary information.

## Code availability

All codes used in this study (experimental codes, numerical optimizer, experimental data) are openly available in Github (https://github.com/DigitalNatureGroup/insitu_optimization) and in Zenodo (10.5281/zenodo.10065462). Please refer to “README.md” file in both repositories for the details regarding the package prerequisite.
