# Supplementary material for: A digital twin approach for experimental acoustic hologram optimization
Source: Commun Eng. 2024 Jan 11;3:12. doi: 10.1038/s44172-024-00160-0 (PMC10955828; doi:10.1038/s44172-024-00160-0)
Supplement: Supplementary file 1 — Supplementary Material [file 44172_2024_160_MOESM1_ESM.pdf]

A digital twin approach for experimental acoustic hologram optimization

Tatsuki Fushimi[1,2*], Daichi Tagami[3], Kenta Yamamoto[3] and Yoichi Ochiai[1,2,4]

[1] Institute of Library, Information and Media Science, University of Tsukuba, Kasuga Campus Kasuga 1-2, Tsukuba, 305-8550, Ibaraki, Japan.
[2] R&D Center for Digital Nature, University of Tsukuba, Kasuga Campus Kasuga 1-2, Tsukuba, 305-8550, Ibaraki, Japan.
[3] Graduate School of Comprehensive Human Sciences, University of Tsukuba, Kasuga Campus Kasuga 1-2, Tsukuba, 305-8550, Ibaraki, Japan.
[4] Pixie Dust Technologies, Inc., Misakicho 2-20-5, Chiyoda, 101-0061, Tokyo, Japan.
*Corresponding author(s). E-mail(s): tfushimi@slis.tsukuba.ac.jp

Experimental Setup

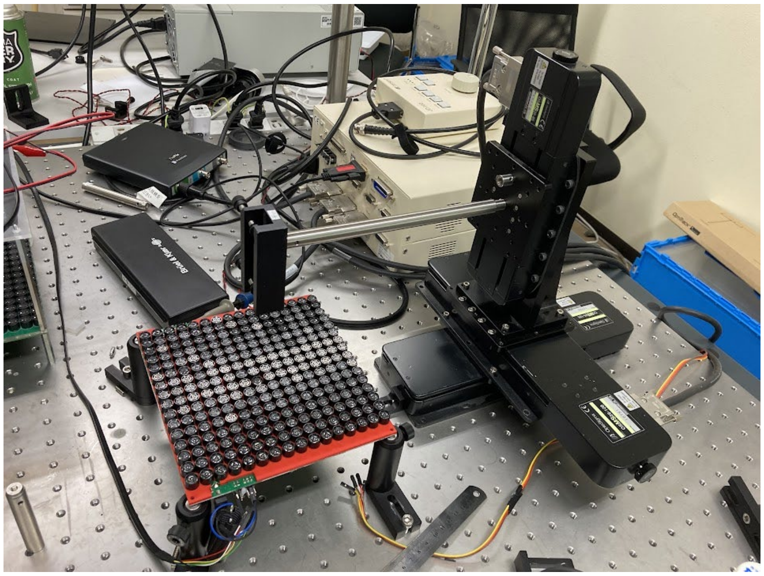

Fig S1: Pressure Measurements
Single Sided 16 by 16 Phased Array Transducers was fixed on top of an optical table (Thorlabs B90120A, SDP90120). Three OptoSigma stages were combined together to form XYZ stage. Calibrated microphone would be attached to the XYZ stage using the holder as shown in the figure and was moved accordingly using the stage.

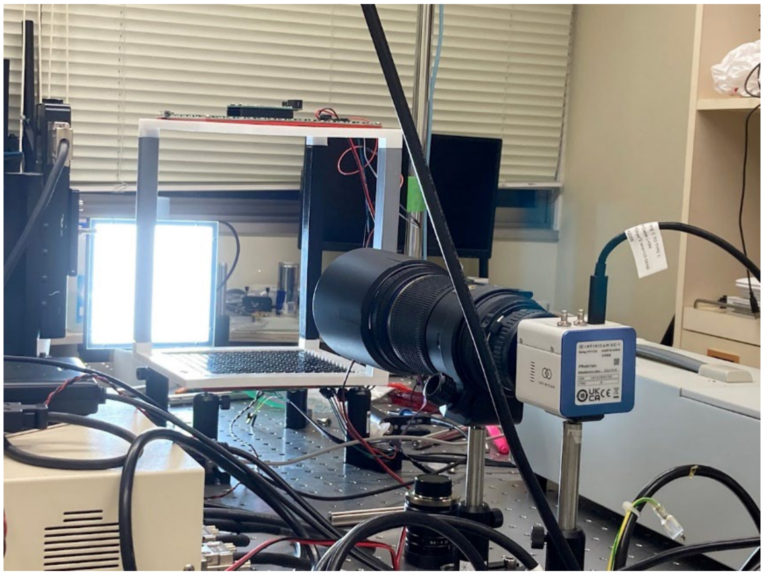

Fig S2: Equilibrium Points Measurements
Double Sided 16 by 16 Phased Array Transducers was fixed on top of an optical table (Thorlabs B90120A, SDP90120). A USB camera with a single-focus lens was used to capture the equilibrium position of the particle, and captured the silhouette of the particle
